# What Is the Effect on Obesity Indicators from Replacing Prolonged Sedentary Time with Brief Sedentary Bouts, Standing and Different Types of Physical Activity during Working Days? A Cross-Sectional Accelerometer-Based Study among Blue-Collar Workers

**DOI:** 10.1371/journal.pone.0154935

**Published:** 2016-05-17

**Authors:** Nidhi Gupta, Marina Heiden, Mette Aadahl, Mette Korshøj, Marie Birk Jørgensen, Andreas Holtermann

**Affiliations:** 1 National Research Centre for the Working Environment, Copenhagen, Denmark; 2 Centre for Musculoskeletal Research, Department of Occupational and Public Health Sciences, University of Gävle, Gävle, Sweden; 3 Research Centre for Prevention and Health, The Capital Region of Denmark, Glostrup, Denmark; 4 Department of Public Health, Faculty of Health and Medical Sciences, University of Copenhagen, Copenhagen, Denmark; 5 Institute of Sports Science and Clinical Biomechanics, University of Southern Denmark, Odense, Denmark; University of the Balearic Islands, SPAIN

## Abstract

**Introduction:**

The aim of the study was to investigate if (a) substituting total sedentary time or long sedentary bouts with standing or various types of physical activity and (b) substituting long sedentary bouts with brief sedentary bouts; is associated with obesity indicators using a cross sectional isotemporal substitution approach among blue-collar workers.

**Methods:**

A total of 692 workers from transportation, manufacturing and cleaning sectors wore an Actigraph GT3X+ accelerometer on the thigh for 1–4 working days. The sedentary (sit and lie), standing, walking, and moderate to vigorous physical activity (MVPA) time on working days was computed using validated Acti4 software. The total sedentary time and uninterrupted sedentary time spent in *brief* (≤5 mins), *moderate* (>5 and ≤30 mins), and *long* (>30mins) bouts, were determined for the whole day and during work and non-work time separately. The obesity indicators, BMI (kg/m^2^), waist circumference (cm) and fat percentage were objectively measured. Isotemporal substitution modelling was utilized to determine the linear association with obesity indicators of replacing 30 min of total sedentary time or long sedentary bouts with standing, walking or MVPA and separately replacing 30 min of long sedentary bouts with brief sedentary bouts.

**Results:**

Workers [mean (standard deviation, SD); age = 45.1 (9.9) years, BMI = 27.5 (4.9) kg/m^2^, %BF = 29.6 (9.5), waist circumference = 94.4 (13.0) cm] sat for 2.4 hours (~32% of the measured time, SD = 1.8 hours) across the day during work period and 5.5 hours (~62% of the measured time, SD = 1.5 hours) during non-work period. Most of the sedentary time was accrued in moderate bouts [work = 1.40 (SD = 1.09) hours] during work and in long bouts during non-work [2.7 (SD = 1.4) hours], while least in long sedentary bouts during work [work = 0.5 (SD = 0.9)] and in brief sedentary bouts [0.5 hours (SD = 0.3)] during non-work. Significant associations with all obesity indicators were found when 30 min of total sedentary time or long sedentary bouts were replaced with standing time (~1–2% lower) or MVPA (~4–9% lower) during whole day, work, and non-work periods. The exception was that a statistically significant association was not observed with any obesity indicator when replacing total sedentary time or long sedentary bouts with standing time during the work period. Significant beneficial associations were found when replacing the long sedentary bouts with brief sedentary bouts (~3–5% lower) during all domains.

**Conclusion:**

Replacing total sedentary time and long sedentary bouts, respectively, not only with MVPA but also standing time appears to be beneficially associated with obesity indicators among blue-collar workers. Additionally, replacing long sedentary bouts with brief sedentary bouts was also beneficially associated with obesity indicators. Studies using prospective design are needed to confirm the findings.

## Introduction

The worldwide prevalence of obesity has almost doubled since 1980 [1]. Based on recent estimates in the European Union, 30–70% of adults are obese [2] with particularly high obesity rates among workers in lower socioeconomic groups, such as blue-collar workers [3–6]. Obesity is associated with an increased risk for all-cause mortality [7], the metabolic syndrome, diabetes, cardiovascular diseases, cancer, arthritis and disability [8–10].

One of the potential risk factors for obesity is prolonged *sedentary time* which has increased in western countries with many adults spending as much as 70% of their waking hours sitting [11]. Sedentary time refers to the time spent on activities (i.e., sitting and lying) that do not increase energy expenditure substantially above resting level (1.0–1.5 METs) [12, 13]. Among office workers, high rates of sitting have been observed (on average 75% of working hours using objective, postural-based measures) [14–16]. Among blue collar workers, the sitting time at work is typically lower, but high levels of sedentary time have been observed during non-work time [17, 18]. Also, due to the nature of work tasks (e.g. long transportation, assembly line work or surveillance) and organizational aspects (e.g., low decision latitude) among blue-collar workers, they may have limited autonomy to reduce or break up prolonged sedentary time during work.

Epidemiological studies have found a positive association between total sedentary time and obesity [19–21], even after statistical adjustment for the level of moderate to vigorous physical activity (MVPA) [22–26]. Recent similar studies have also addressed the importance of sedentary time patterns (how sedentary time is distributed across the day) with respect to obesity [27, 28]. Some experimental and epidemiological studies have found long sedentary bouts to be associated with, for example, biomarkers of cardio-metabolic health [29, 30]. In contrast, brief sedentary bouts are not considered a health hazard to the same extent [27–29, 31].

An emerging method to investigate the associations between sedentary time and health outcomes is isotemporal substitution analysis. This method calculates the estimates of the association when replacing time spent on one activity with another, while keeping the total time fixed (isotemporal) [32, 33]. Thus this method helps to answer a very important question, how to spend our discretionary time per day for minimizing health risk and achieving optimum health. It has currently been used to explore the association between various types of physical activity or sedentary behaviors and depression, cardiovascular disease, and weight gain [30, 32, 34, 35]. This question cannot be answered with statistical models which adjust for classical confounders and wear time (i.e., ‘single activity’ models) or models which also adjust for other sedentary behavior and physical activities (i.e., ‘partition’ models) as described in a study by Mekary et al [33]. In the study by Healy et al [30], the sedentary time patterns (e.g., prolonged and non-prolonged sedentary bouts) were investigated among adult patients with type 2 diabetes, but not in specific working populations, such as blue-collar workers. The isotemporal substitution analysis is particularly interesting in this population as it provides theoretical estimates of the benefits of “rotating” job tasks and thereby shifting exposures among sedentary and various types of physical activity.

A limitation of previous studies using the isotemporal substitution approach is the use of count-based accelerometer thresholds to determine sedentary time [30]. Both location of wear (for example hip) and usage of count-based accelerometer thresholds have been criticized for their inability to accurately differentiate sedentary postures from standing [36]. Both of these limitations may lead to incorrect information about sedentary time patterns [37] and thus should not be used to investigate the effect of replacing the sedentary time with standing time on various health outcomes. Additionally, previous studies using the isotemporal substitution analysis to explore the relationship between various types of physical activity/sedentary behavior and health outcomes have almost exclusively focused on whole day measurements [30, 35]. It has been shown that work and non-work time differ in terms of sedentary patterns [38, 39] and also in terms of the barriers and facilitators for modifying these patterns. Thus, it is important to understand the contribution of each of these domains to the overall risk of obesity. Previous studies [40] have also not investigated the effect of replacing sedentary time with a *detailed spectrum* of physical activities (i.e., standing, walking, and MVPA instead of standing and stepping only) on obesity.

Thus, the aim of this study was to investigate the association of obesity indicators with total sedentary time and bouts of sedentary time (brief, moderate and long sedentary bouts) during whole day, work and non-work time among blue-collar workers. Furthermore, these associations were further explored when replacing total sedentary time and long sedentary bouts, respectively, with various types of physical activity, as well as replacing long sedentary bouts with brief sedentary bouts.

## Material and Methods

### Study design and population

Our study is a part of the Danish PHysical ACTivity cohort with Objective measurements (DPhacto). The main aim of DPhacto is to investigate the association between objectively measured physical activities at work and development of musculoskeletal pain among blue-collar workers [41]. A detailed recruitment of the study population, inclusion and exclusion criteria and data collection is reported elsewhere [41].

This study includes the cross-sectional baseline data of DPhacto, collected from spring 2012 to spring 2014 at workplaces engaged in three different occupational sectors (cleaning, transport and manufacturing) in Denmark. In total, 2107 eligible workers from 15 companies were recruited in collaboration with a large labor union between December 2011 and March 2013. Workers were excluded if they were in a white-collar occupation (managers/administrative workers), pregnant, had a fever, or a band aid allergy.

All workers provided their written informed consent prior to participation. The present study was conducted according to the Helsinki declaration and approved by the Danish data protection agency and local Ethics Committee (The Capital Region of Denmark, H-2-2012-011).

### Data collection

All eligible blue-collar workers responded to a questionnaire about socio-demographic measures, lifestyle and health-related behaviours and participated in measurements of obesity indicators. Additionally, they were equipped with an Actigraph accelerometer (Actigraph GT3X+, Actigraph LLC, Florida, USA) placed on the right thigh [42] for four consecutive days (4 x 24 hours), including at least two working days to determine their sedentary and physical activities spectrum [41]. They were also equipped with a short paper diary concerning their working hours, non-work time, time in bed (going to bed, to sleep, and getting out of bed), non-wear time, and time of reference measurement every day during the measurement period. Detailed pre-instructions given to the workers are mentioned elsewhere [17, 41, 43].

#### Objective measurements of sedentary time and types of physical activity

The sedentary time and various types of physical activity were determined using a custom-made MATLAB program (Acti4), estimating time spent in various types of physical activity [moving (upright position that could not be classified as standing still or walking or running; for example, very small steps on the same spot), walking, running, stair climbing, and cycling] and body postures (sitting, lying and standing still) across the day(s) with a sensitivity and specificity of more than 94% and 99%, respectively, during semi-standardized conditions [42]. During free living conditions (around 140 hours of measurements), the sensitivity and specificity of the Acti4 program for detecting the sitting posture was 98% and 93%, compared to pressure sensors placed in the back pockets [42].

Briefly, accelerometer data were low-pass filtered with a 5Hz 4^th^ order Butterworth filter and split-up into 2 sec intervals with 50% overlap. Then, the occurrence of sedentary posture was defined as the posture in which the inclination of the thigh is above 45°. The daily reference measurements (i.e. standing in an upright position for 15sec on every measured day) were used to obtain the angle between the leg and accelerometer axis. The technical definitions of the identification of other activities using Acti4 program are reported in detail elsewhere [42].

Using the diary information about the start and end time of work and the time in and out of bed at night, the work and non-work periods were defined. All non-working days and non-wear periods were excluded according to previously reported criteria [17, 43]. As this study was intended to address working days only, only days with objective measurements during the working periods were included. Further, only workers with at least one valid day comprising at least 10 hours of wear time during waking hours were included in the analysis on whole day [17, 43, 44]. For the specific analyses of sedentary time during work and non-work domain, workers with at least one day of valid measurement of work and non-work time period were included; (a) a valid work time period: at least 4 hours of work time or 75% of the individual’s average reported working time, and (b) valid non-work time period: at least 4 hours of non-work time or 75% of the individual’s average reported non-work time [17, 43]. A day consisted of 24 hours starting from midnight to midnight, while whole day was defined as all waking hours during a day. Work was defined as the self-reported hours spent at the primary occupation, and non-work time was defined as the remaining time except time in bed during a day.

Subsequently, based on the measured periods of sedentary time on working days, total sedentary time (total time spent sitting or lying) in the following domains were retrieved; (a) whole day (total measured sedentary hours during the entire measurement period divided by the number of days), (b) work (total measured sedentary hours during working periods divided by the number of days) and (c) non-work time (total measured sedentary hours except time in bed during non-work time periods divided by the number of days). Total time periods spent ‘standing still’ and ‘moving’ were merged and divided by the number of days measured to calculate ‘standing time’. Total time periods spent ‘stair-climbing’, ‘running’ and ‘cycling’ were merged and divided by the number of measured days to calculate MVPA time. Total time spent walking was divided by the number of days to calculate ‘walk time’. ‘Time in bed’ was estimated based on the self-reported time in the diary for ‘going to bed to sleep and getting out of bed’ and was averaged across the days measured. Total wear/measured time during whole day and separately during work and non-work period were calculated by adding the registered measured time (excluding non-recorded periods which were less than ~1% of the total measured time) from accelerometers in each domain on all days divided by the number of measured days.

*Exposure Variation Analysis of sedentary time*. The sedentary time patterns were determined using Exposure Variation Analysis (EVA; [45]). Based on previous studies [16, 30, 39, 46], EVA was utilized to categorize uninterrupted sedentary bouts according to their duration, ‘long bouts’ (LB) (average time/day spent in uninterrupted sedentary bouts >30 min), ‘moderate bouts’ (MB) (average time/day spent in uninterrupted sedentary bouts >5 and ≤30min), and ‘brief bouts’ (BB) (average time/day spent in uninterrupted sedentary bouts ≤5mins) during whole day, work and non-work time [39].

#### Objective measurement of obesity indicators

The weight, body fat percentage (Tanita, model BC418 MA) [47], and waist circumference of the workers were objectively measured. The waist circumference with millimeter precision was measured horizontally midway between the top edge of the hip and lower ribs using a measurement tape (Seca, model 201) [48]. The average of the two measurements was recorded. Height was measured without shoes using a stadiometer (Seca, model 213) to the nearest 0.1 cm while weight and fat percentage (%BF) were measured without shoes and socks using the Tanita (model BC418 MA) bio-impedance segmental body composition analyzer [47], to the nearest 0.1 kg and 0.1%, respectively. Their BMI (kg/m^2^) was calculated as weight (kg) divided by height (m) squared.

#### Potential confounders

The confounders were chosen *a priori* based on theoretical considerations and previous studies investigating the association between sedentary time and obesity [26, 27, 30, 49]. Sex and age of the workers were determined from each worker’s unique Danish civil registration number. Smoking behavior was determined from the question: “Do you smoke?” with 4 response categories summarized into three groups; smokers (yes daily; yes sometimes), ex-smokers (used to smoke, but not anymore) and non-smokers (have never smoked). Alcohol intake was determined by the item ‘How much alcohol did you drink during the last week’ with responses in number of units per week. Poor dietary habits were determined by averaging the responses of the following two items ‘How often do you usually eat/drink candy, ice cream, chocolate, soft drinks’ and ‘fastfood, pizza, burger, shawarma, etc.’ with four responses (daily, 3-4/week, 1-2/week, and rarely). Influence at work (decision authority) was determined by the 2-item scale from the Copenhagen Psychosocial Questionnaire [50]. The responses were scored on a Likert scale with response categories ranging from 0 (never) to 5 (always). A composite scale measuring influence at work was constructed by calculating the mean rating of both items. For the analysis, this scale was recoded into a 0–100 scale, where a larger score represented a higher degree of influence at work [17]. All confounders were treated as continuous variables except smoking behavior and sex which were treated as categorical variables.

### Statistics

All statistical analyses described below were performed for each of the three time domains, i.e., whole day, work and non-work time; and for each of the three indicators of obesity, i.e., BMI, %BF and waist circumference. The results of the associations between sedentary time variables and obesity indicators were presented as unstandardized regression coefficients per 30 minutes of modelled exposure. All models were adjusted for the potential confounders age, sex (reference: male), smoking (reference: non-smokers), alcohol intake, and dietary habits. The associations were tested using three linear regression models: ‘the single activity model’, ‘the partition model’ and ‘the isotemporal substitution model’.

First, four independent single activity models were fitted to assess the association between each independent sedentary time variable (i.e., total sedentary time and long, moderate and brief bouts) and each of the dependent obesity indicators using ordinary linear least-squares regression analysis, adjusted for total measured time in addition to the abovementioned confounders.

Second, the partition models were fitted by adjusting the single activity models for standing, walking, and MVPA time instead of total measured time. In the models containing long, moderate or brief sedentary bouts, these variables were entered simultaneously.

Third, the isotemporal substitution models were fitted to test the association with obesity indicators when replacing total sedentary time or long sedentary bouts (>30 minutes/day) with an equal amount of time spent on another activity (+30 min/day of standing, walking or MVPA time). Similarly, the effect on the indicators of obesity of replacing long sedentary bouts with short sedentary bouts was also estimated. In these models, all independent variables, except the variable of interest (total sedentary time or long sedentary bouts), plus the total measured time and confounders were entered into the model simultaneously. Thus, the regression coefficient of an included variable (brief sedentary bouts, standing, walking or MVPA) in the model represented the population-based average change in the indicators of obesity due to increasing 30 minutes/day of that variable and decreasing the 30 minutes/day of the variable of interest (total sedentary time or long sedentary bouts), keeping the time in all other modelled activities constant.

In all models, time spent on each activity in both domains was entered. The reason for this is that the time of activities in the domains are correlated [51] and therefore needs to be mutually adjusted for by being simultaneously entered in the model. To avoid overfitting, the combined time spent on activities in both domains except the variable of interest (i.e., total sedentary time or long sedentary bouts) was entered in the models.

The sensitivity of the results obtained from the isotemporal models was tested by; (1) additionally adjusting for influence at work, (2) additionally adjusting for the self-reported ‘time in bed’, and (3) additionally adjusting for shift work and job type of the workers in the primary analysis model.

In the analyses, no major multi-collinearity issues were detected (tolerance index >0.20, VIF values <5) [52]. Visual inspection of P-P plots, histograms of standardized residuals, and scatterplots of standardized residuals against standardized predicted values indicated that assumptions of linearity were fulfilled, and that residuals were normally distributed and homoscedastic. Two outliers, indicated in boxplots, were removed from the analyses on waist circumference due their effect on the model fit.

Using the sample size calculation for linear regression, inclusion of at least 398 workers were sufficient to detect, with statistical power of 80% and alpha of 5%, a slope of 1 cm in waist circumference, 0.35 kg/m^2^ in BMI, and 0.40% in fat percentage attributed to 30minutes/day of total sedentary time and long sedentary bouts, assuming a standard deviation (SD) of 3 units per 30minutes/day. We considered these slopes to be meaningful based on previous studies [30, 53].

## Results

**Fig 1:**
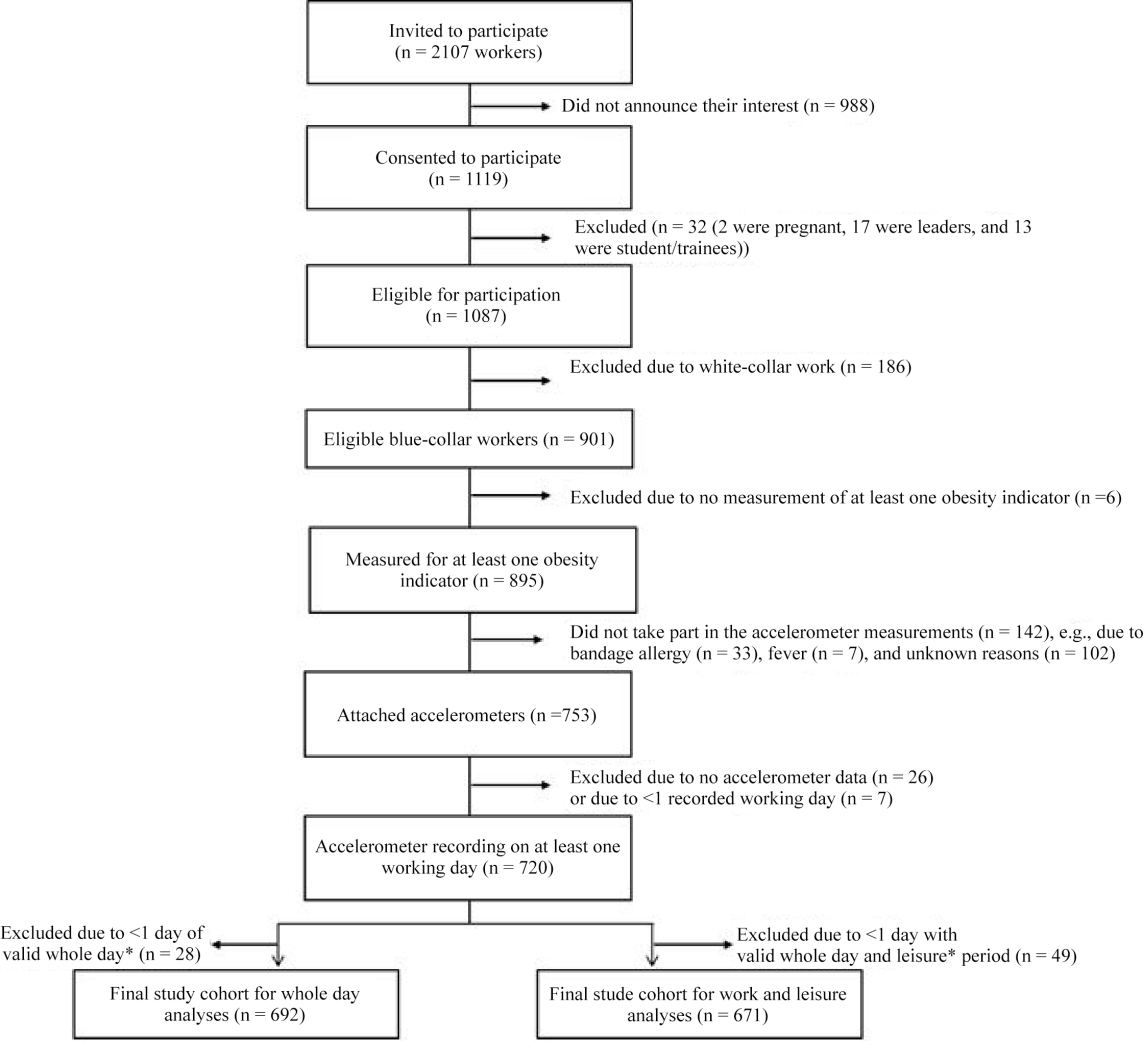
Recruitment flow of the study population. Note: *Valid whole day, work and non-work time are defined in the ‘Material and Methods’ section.

Fig 1 illustrates the flow diagram of the participants in the study. In total, 2107 workers were invited to participate of whom 901 blue-collar workers were eligible for participation. Of them, 692 were included in the analysis on whole day while 671 were included in the analysis of the work and non-work time domain.

For the analyses of whole day, work and non-work time, ~32,334 (2,023 valid days), ~13,521 (1,774 valid days) and ~15,550 (1,774 valid days) valid hours were measured, respectively. Participant characteristics and accelerometry-based measurements are described in Tables 1 and 2. Workers spent more time being sedentary during non-work time than during work. Overall during the whole day and non-work domain, they spent the majority of time being sedentary and the least time in MVPA. In the work domain, most time was spent in standing and least in MVPA. More specifically, during non-work time, the majority of the sedentary time was spent in long bouts and the least time in short bouts. During work time, the majority of the sedentary time was spent in moderate bouts and the least time in long bouts. Workers self-reported, on average, 5.9 (SD = 1.5) hours in bed per day (duration between going to and getting out of bed).

**Table 1:** Participant characteristics and accelerometer-based measurements of the blue-collar workers involved in whole day analyses (n = 692) in the Danish PHysical ACTivity cohort with Objective measurements (DPhacto).

Variables	N	%	Mean	SD
Age (years)	692		45.1	9.9
Sex				
Males	371	54		
Females	321	46		
Working sector				
Cleaning	137	20		
Manufacturing	487	70		
Transport	67	10		
Shift work				
Fixed day work	529	79		
Fixed night/night shift work	101	15		
Other^∞^	41	6		
Height (cm)	682		172.0	10.1
Weight (kg)	692		75.8	13.5
BMI (kg/m^2^)	682		27.5	4.9
Normal (<25)	227	33		
Overweight (25–29.9)	269	40		
Obese (≥30)	184	27		
Poor dietary habits (1–4)^±^	678		2.2	1.0
Smoking status				
Smokers	261	31		
Ex-smokers	206	31		
Non-smokers	207	39		
Alcohol intake (units/week)	646		4.5	6.6
Fat percentage	682		29.6	9.5
High risk^҂^	335	49		
Waist circumference (cm)	477		94.4	13.0
High risk^ǂ^	210	44		
Influence at work (0–100%)^≠^	485		62.0	26.6

BMI = body mass index

^±^1 = eating fast food every day and 4 = eating fast food rarely

^≠^0 = low influence, 100 = high influence

^∞^includes other type of work such as rotating shiftwork which is not fixed, fixed evening shift work etc

^҂^based on the threshold of high risk due to high body fat percentage based on sex and age

^ǂ^high risk = waist circumference is >102 cm for males and >88 cm for females.

**Table 2:** Accelerometer-derived sedentary time (time spent lying or sitting) variables during whole day (n = 692), work (n = 671) and non-work (n = 671) time domain among blue-collar workers in the Danish PHysical ACTivity cohort with Objective measurements (DPhacto).

	Whole day		Work		Non-work	
Variables	Mean	SD	Mean	SD	Mean	SD
Total sedentary time (hours/day)	7.83	2.13	2.45	1.75	5.49	1.46
LB (hours/day)	3.17	1.67	0.50	0.94	2.65	1.40
MB (hours/day)	3.60	1.28	1.40	1.09	2.30	0.80
BB (hours/day)	1.06	0.58	0.55	0.50	0.54	0.25
Total measured time*	15.93	1.45	7.60	1.29	8.79	1.60
Number of valid days	2.92	0.96	2.64	0.95	2.64	0.95
Standing time (hours/day)	6.07	1.83	3.88	1.56	2.46	0.97
Walking time (hours/day)	1.85	0.61	1.20	0.50	0.72	0.33
MVPA time (hours/day)	0.18	0.21	0.06	0.07	0.12	0.19

LB = long sedentary bouts (average time/day spent in uninterrupted sedentary bouts >30 min), MB = moderate sedentary bouts (average time/day spent in uninterrupted sedentary bouts >5 and ≤30min), BB = brief sedentary bouts (average time/day spent in uninterrupted sedentary bouts ≤5mins), MVPA = moderate to vigorous physical activity.

* total measured time and the time in bed do not sum up to 24 hours due to the inclusion of days with at least 10 hours of measured waking time but not all waking hours on that day.

The results of the *single activity* models (shown in S1 Table) showed that total sedentary time was significantly positively associated with higher obesity indicators during whole day and non-work time, but not during work time. Similar results were observed for LB, while BB was significantly negatively associated with lower obesity indicators in all domains. No significant associations between MB and obesity indicators were found for any domain.

When adjusted also for other sedentary behavior and physical activities in the model, beside classical confounders (see Methods section), the results of the *partition* models (shown in S2 Table) showed similar results to the single activity models for all analyses. However, the association between total sedentary time and %BF did not remain significant during whole day [B(95%CI) = 0.09(-0.11 to 0.29) %, P = 0.36] and non-work time [B(95%CI) = 0.19(-0.04 to 0.43) % P = 0.11]. Moreover, the association between brief sedentary bouts and obesity did not remain significant for BMI during work [B(95%CI) = -0.34(-0.77 to 0.08) kg/m^2^ P = 0.11].

Table 3 shows the results of the *isotemporal substitution analyses* indicating the ‘substitution association’ with obesity indicators when replacing 30 minutes/day of sedentary behaviors with 30 minutes/day of other activities during the whole day. Table 4 shows the corresponding results for work and non-work time domains separately. The estimates of the obesity indicators decreased significantly when total sedentary time or long sedentary bouts were replaced with standing time (~1–2% lower) or MVPA (~4–9% lower; equivalent to a meaningful amount of ~3.8–4.2 cm waist circumference, ~1.9–2.7% fat percentage and ~1.1–1.4 kg/m^2^ BMI per 30 minutes/day) during whole day and non-work time. Similar results were obtained when long sedentary bouts were replaced with brief sedentary bouts (~3–5% lower; equivalent to a meaningful amount of ~2.6–2.7 cm waist circumference, ~1.4–1.6% fat percentage, and ~0.8–0.9 kg/m^2^ BMI per 30 minutes/day) during whole day and non-work time. During work time, similar results were observed for brief sedentary bouts (~3–4% lower) and MVPA (~3–6% lower), but not for standing time. Interestingly, replacing total sedentary time or long sedentary bouts in any domain with increased walking time was not significantly associated with any of the indicators of obesity.

**Table 3:** Results of the isotemporal substitution analyses^*^ indicating the association with obesity indicators when replacing sedentary time (-30 minutes/day) with an equal amount of time of another sedentary pattern or physical activity (+30 minutes/day) during whole day among blue-collar workers (n = 692) from Danish PHysical ACTivity cohort with Objective measurements (DPhacto).

Reduced	Increased	B (95%CI)	P	B (95%CI)	P	B (95%CI)	P
		Waist circumference (cm)		Fat percentage (%)		BMI (kg/m^2^)	
Total sedentary time	Stand	**-0.50(-0.81 to -0.18)**	**0.00**	**-0.29(-0.45 to -0.13)**	**0.00**	**-0.17(-0.28 to -0.06)**	**0.00**
	Walk	0.40 (-0.53 to 1.33)	0.40	0.19(-0.26 to 0.65)	0.41	0.17(-0.15 to 0.48)	0.29
	MVPA	**-4.21 (-6.94 to -1.47)**	**0.00**	**-2.70(-4.03 to -1.37)**	**0.00**	**-1.37(-2.29 to -0.44)**	**0.00**
LB	Stand	**-0.86(-1.22 to -0.5)**	**0.00**	**-0.46(-0.65 to -0.27)**	**0.00**	**-0.31(-0.44 to -0.17)**	**0.00**
	Walk	0.57(-0.35 to 1.5)	0.22	0.27(-0.19 to 0.72)	0.25	0.18(-0.14 to 0.5)	0.26
	MVPA	**-3.93(-6.62 to -1.23)**	**0.00**	**-2.38(-3.7 to -1.06)**	**0.00**	**-1.28(-2.2 to -0.35)**	**0.01**
	MB	-0.36(-0.89 to 0.16)	0.18	-0.10(-0.38 to 0.17)	0.46	-0.16(-0.35 to 0.03)	0.09
	BB	**-2.60(-3.55 to -1.65)**	**0.00**	**-1.43(-1.92 to -0.94)**	**0.00**	**-0.82(-1.17 to -0.48)**	**0.00**

LB = long sedentary bouts (average time/day spent in uninterrupted sedentary bouts >30 min), MB = moderate sedentary bouts (average time/day spent in uninterrupted sedentary bouts >5 and ≤30min), BB = brief sedentary bouts (average time/day spent in uninterrupted sedentary bouts ≤5mins), MVPA = moderate to vigorous physical activity

*adjusted for age, sex, smoking status, alcohol intake, dietary patterns, and total measured time; estimates in bold are significant at p <0.05.

**Table 4:** Results of the isotemporal substitution analyses^*^ indicating the association with obesity indicators when replacing sedentary time (-30 minutes/day) with an equal amount of time of another sedentary pattern or physical activity (+30 minutes/day) during work and non-work domain among blue-collar workers (n = 671) from Danish PHysical ACTivity cohort with Objective measurements (DPhacto).

Reduced	Increased	B(95%CI)	P	B(95%CI)	P
		Work		Non-work	
Waist Circumference (cm)					
Total sedentary time	Stand	-0.24(-0.6 to 0.11)	0.18	**-0.82(-1.26 to -0.37)**	**0.00**
	Walk	0.55(-0.38 to 1.48)	0.24	-0.02(-1.01 to 0.97)	0.97
	MVPA	**-3.43(-6.25 to -0.61)**	**0.02**	**-4.00(-6.75 to -1.26)**	**0.00**
LB	Stand	**-0.59(-1.16 to -0.03)**	**0.04**	**-0.94(-1.39 to -0.49)**	**0.00**
	Walk	0.67(-0.36 to 1.7)	0.20	0.33(-0.62 to 1.28)	0.50
	MVPA	**-3.42(-6.3 to -0.55)**	**0.02**	**-3.77(-6.51 to -1.03)**	**0.01**
	MB	-0.08(-0.81 to 0.66)	0.84	-0.42(-0.97 to 0.14)	0.14
	BB	**-2.40(-3.43 to -1.36)**	**0.00**	**-2.74(-3.77 to -1.72)**	**0.00**
Fat percentage (%)					
Total sedentary time	Stand	-0.14(-0.32 to 0.04)	0.12	**-0.42(-0.65 to -0.19)**	**0.00**
	Walk	0.27(-0.19 to 0.74)	0.25	0.00(-0.49 to 0.49)	1.00
	MVPA	**-1.91(-3.29 to -0.52)**	**0.01**	**-2.18(-3.53 to -0.84)**	**0.00**
LB	Stand	**-**0.17(-0.48 to 0.13)	0.27	**-0.55(-0.78 to -0.32)**	**0.00**
	Walk	0.47(-0.06 to 0.99)	0.08	0.09(-0.38 to 0.55)	0.72
	MVPA	**-1.50(-2.9 to -0.11)**	**0.03**	**-1.88(-3.22 to -0.55)**	**0.01**
	MB	0.20(-0.19 to 0.6)	0.32	-0.18(-0.46 to 0.1)	0.21
	BB	**-1.22(-1.76 to -0.68)**	**0.00**	**-1.60(-2.11 to -1.09)**	**0.00**
BMI (kg/m^2^)					
Total sedentary time	Stand	-0.08(-0.2 to 0.05)	0.24	**-0.24(-0.4 to -0.08)**	**0.00**
	Walk	0.25(-0.07 to 0.57)	0.12	0.08(-0.25 to 0.41)	0.64
	MVPA	**-1.03(-1.97 to -0.09)**	**0.03**	**-1.20(-2.12 to -0.28)**	**0.01**
LB	Stand	-0.17(-0.38 to 0.04)	0.11	**-0.32(-0.48 to -0.16)**	**0.00**
	Walk	0.28(-0.08 to 0.64)	0.13	0.13(-0.19 to 0.45)	0.43
	MVPA	**-0.96(-1.92 to 0.00)**	**0.05**	**-1.10**(-2.02 to -0.18)	**0.02**
	MB	-0.02(-0.29 to 0.26)	0.90	-0.16(-0.36 to 0.03)	0.10
	BB	**-0.72(-1.1 to -0.35)**	**0.00**	**-0.87(-1.22 to -0.52)**	**0.00**

LB = long sedentary bouts (average time/day spent in uninterrupted sedentary bouts >30 min), MB = moderate sedentary bouts (average time/day spent in uninterrupted sedentary bouts >5 and ≤30min), BB = brief sedentary bouts (average time/day spent in uninterrupted sedentary bouts ≤5mins), MVPA = moderate to vigorous physical activity

*adjusted for age, sex, smoking status, alcohol intake, dietary patterns, and total measured time; estimates in bold are significant at p <0.05. Time spent on each activity except the activity of interest (total sedentary time and LB) is the combined time spent during both domains.

### Results of the sensitivity analyses

Adjustment for influence at work did not change the estimates of the primary analyses of replacing 30 minutes of total sedentary time with standing, walking or MVPA. Similar results were observed for replacing long sedentary bouts with standing, walking, MVPA or brief sedentary bouts, except that the results of replacing 30 minutes of long sedentary bouts during work with MVPA became marginally non-significant for %BF [B(95%CI); -1.42 (-3.02 to 0.19)%, P = 0.08) and BMI (-1.03(-2.15 to 0.09) kg/m^2^, P = 0.07) (results not shown).

Similar estimates to the primary analysis using isotemporal substitution were also observed when adjusting for time in bed (results not shown), except that the results of replacing 30 min of long sedentary bouts during work with MVPA became marginally non-significant [-0.92(-1.88 to 0.05) kg/m^2^, P = 0.06] for BMI.

Adjustment for job type and shift work did not change the estimates of the primary analyses using isotemporal substitution, except that the results of replacing total sedentary time with standing became significant [-0.26(-0.46 to -0.06) %, P = 0.01] for fat percentage and replacing long sedentary bouts with standing became non-significant [-0.45 (-1.15 to 0.25) cm, P = 0.21] for waist circumference during work.

## Discussion and Conclusion

The most important finding of the present cross-sectional study is that among blue-collar workers beneficial associations with indicators of obesity were observed by theoretically replacing time spent on sedentary behavior with standing or higher intensity physical activity. Beneficial findings were also observed when time in long sedentary bouts was reallocated to short sedentary bouts.

The amount of sedentary time in this study was similar to other studies among blue-collar workers [17, 18, 39, 43]. The workers sat for ~50% of their waking time with more sedentary time being spent during non-work (~62% of total non-work time) than work (~32% of the working time). This might reflect a compensation for not having sufficient opportunities to recover from strenuous work tasks. During whole day, workers spent the most time in long and moderate sedentary bouts and the least in brief sedentary bouts. They spent about 38% of the time standing (with ~25% standing still), ~12% walking and ~1% MVPA, which compares well with previous research on blue-collar workers [39] except for walking being slightly lower in this population (~26% in the previous study [39]).

Workers who not only spent more time in long, but also less time in brief sedentary bouts had higher indicators of obesity and vice versa, even after adjusting for potential confounders and wear time (the single activity model) and other activities performed (the partition model). For example, spending 30 minutes more in long sedentary bouts during non-work time was associated with 0.98 cm (~1%) increase in waist circumference, while spending 30 minutes more in brief sedentary bouts during non-work time was associated with a decreased waist circumference by 2.94 cm (~3%) (see S2 Table), keeping the other sedentary behavior and physical activities constant. The results of brief sedentary bouts correspond well with previous studies on the association between breaks in prolonged sitting (e.g., transitions from a sedentary to an active state lasting ≥1min) and obesity indicators [27, 28]. The brief sedentary bouts could be considered as a ‘proxy’ for ‘breaking up’ long sedentary bouts by various physical activities (i.e., intermittent physical activities). It is possible that frequent interruptions of prolonged sedentary bouts facilitate lipid metabolism and glucose removal from the blood due to intermittent muscle contractions [31, 54], which may, in the long term, decrease the probability of becoming obese. However, this hypothesis requires further investigation on metabolic variables in longitudinal studies. Nevertheless, these results suggest that the distribution of sedentary time patterns, in addition to total sedentary time, may be important for obesity.

During work, however, significant associations between total sedentary time or long sedentary bouts and obesity indicators were not observed, which is in contrast to some studies [55–58], but not all [59, 60]. One explanation for the conflicting results could be differences in the study population and types of measures used in the studies. In studies where a significant positive association between sedentary time at work and obesity have been observed, workers were very sedentary at work (sat for as much as 6 hours during work or 60% of the working time) compared to our study [55, 57]. Also most of these studies used self-reported measures (telephone interviews [55] and questionnaires [56–58]) to assess sedentary time, which could lead to misclassification of the workers’ occupational sedentary time [61] due to reporting bias.

Our findings suggest that replacing 30 minutes of total sedentary time or long sedentary bouts with 30 minutes of MVPA during a day has a considerable beneficial meaningful association with obesity during all domains, which confirms the previously established beneficial role of MVPA in prevention and management of obesity [62, 63]. However, beneficial associations were also found when total sedentary time and long sedentary bouts, respectively, were replaced with standing, and when long sedentary bouts were replaced with brief sedentary bouts. Although, these results on replacing sedentary time with standing time are not considered clinically meaningful in our study, they are consistent with previous studies [30], where replacing long sedentary bouts with light physical activity during whole day resulted in lower BMI [30] and waist circumference [32]. During work, these results were similar except for replacing total sedentary time or long sedentary bouts with standing time during a day. Some previous studies have indicated a beneficial effect of standing at work [64–67]. However, most of these studies were either conducted in office workers only [65] or utilized self-reported standing measures [66]. Therefore, we need future studies to verify the association between standing at work and obesity among blue-collar workers.

Replacing prolonged sedentary time with brief sedentary bouts or standing may be more feasible than replacing it with MVPA among blue-collar workers who are already engaged in highly strenuous work tasks. We estimated that replacing ~45 minutes of long sedentary bouts with brief sedentary bouts or ~137 mins of long sedentary bouts with standing will have equal effect on obesity as replacing 30 min of long sedentary bouts with MVPA. In relative terms, ~45 min increase in brief sedentary bouts and ~137 min increase in standing corresponds to 69% or 37% increase in daily time spent in these activities, and may be more feasible among blue-collar workers than increasing 30 minutes in MVPA which corresponds to a 250% increase in their daily MVPA. Thus, the replacement of sedentary time with other activities such as standing and brief sedentary bouts may be relevant and practical in this cohort as long as the sufficient time is reallocated. Nevertheless, the observed results suggest that even a modest change such as replacing 30 minutes of long sedentary bouts with standing or brief sedentary bouts may reduce obesity among blue-collar workers. However, for these workers who may require sedentary time as ‘rest’ period between high strenuous working tasks, it might be appropriate to shift between job tasks to ensure reduction in prolonged, unbroken sedentary bouts instead of aiming to reduce total sedentary time. More research is needed to determine which activity-replacement, in practice, is most feasible for achieving sustainable favorable changes in obesity among blue-collar workers without affecting their work demands-recovery balance.

Interestingly, we did not find any beneficial association with obesity indicators of replacing total sedentary time or long sedentary bouts with walking time. These findings are in contrast to a growing literature on the positive effect of light physical activities on health outcomes including obesity [30, 35]. Indeed, previous studies using isotemporal substitution analysis have shown that walking or light physical activities are important for health outcomes such as weight, BMI [30], waist circumference [30] and cardiovascular diseases [32] among diabetic adults or the general population. The findings are intriguing, and should be investigated further among blue-collar workers specifically.

### Strength and limitations and methodological considerations

A major strength of our study is the study population of blue-collar workers, being homogeneous with respect to being employed in blue collar work, but offering a great variation in sedentary time and various types of physical activity. We also used the validated software, Acti4 to assess the exposure. It is capable of discriminating between different types of physical activity and body postures with excellent sensitivity and specificity [42]. Previous studies using isotemporal substitution analysis have recommended this method which is based on posture identification instead of count-based accelerometer thresholds for calculating objectively measured sedentary and physical activity time [30]. Another strength of the study was the separation between prolonged and brief sedentary bouts using exposure variation analysis (EVA). EVA is a versatile generic approach for quantifying the level and frequency of activities, as demonstrated by the use of EVA for analyses of, e.g. muscle activity [68], working postures [69] and physical activity intensities [46]. Although we did not have objective measurements of sleep duration, we adjusted for self-reported time in bed which did not substantially change the overall results of the primary analyses.

The main limitation of the study is the cross-sectional study design, which does not allow for inferences about causal associations with obesity of replacing prolonged sedentary activities with brief sedentary bouts and various types of physical activity. Thus, future prospective studies are needed to assess the strength and direction of this association in different domains. Since our study included a sample of Danish blue-collar workers only, our results may not be generalized to the general population of blue-collar workers in Denmark, let alone in industrialized countries in general. Furthermore, the results may be affected by residual confounding from medication usage, prevalence of chronic diseases, working status, family history, and activity patterns during commuting to work. The adjustment for influence at work was only performed in the sensitivity analyses due to a large amount of missing values in the variable (~30%), although adjusting for this variable did not change the results of the primary analysis. Another limitation is that the use of traditional statistical analyses may not be suitable due to the constrained nature of the data used in this study [70]. This requires future investigation using compositional data analysis, specially designed for constrained data [70], to verify the results obtained in our study. Although adjustment for other health risk behaviors such as poor diet, smoking, and high alcohol intake did not change the results obtained in our study, these health risk behaviors were common in this cohort and thus future studies should investigate the association of these risk factors, beside activity levels, which may also be associated with obesity among these workers. Although, defining a day from midnight to midnight is a common practice in previous research [71], it may have some consequences on the overall distribution and amount of time spent sedentary and in physical activities. This is reflected in the short average ‘time in bed’ duration in this sample, where time in bed on the last measured day is stopped at midnight. Thus, future studies using some other definitions of a ‘day’ are needed to verify the results of our study. It is also possible that the observed strength of the associations between sedentary behaviors and indicators of obesity are different within different categories of obesity. However such analysis requires a large sample size and thus warrants a future investigation. The fluctuation or variation in work and non-working hours within a day could also contribute to the obesity among these workers which needs to be investigated upon in future studies.

In conclusion, total sedentary time and uninterrupted long sedentary bouts were positively associated with obesity indicators during whole day and non-work time while brief sedentary bouts were negatively associated with obesity indicators during all domains. Our findings indicate that beneficial health effects may be gained by replacing total sedentary time and uninterrupted long sedentary bouts not only with MVPA, but also with standing among blue-collar workers. Similar benefits may also be gained by replacing uninterrupted long sedentary bouts with brief sedentary bouts among these workers. However, these interpretations of the results need to be confirmed in future studies using a prospective study design.

## Supporting Information

S1 Dataset This dataset contains two data files, ‘*whole day*’ and ‘*work and non-work*’. The data file ‘whole day’ contains data for investigating the association between sedentary patterns during whole day and obesity indicators (N = 692) and the data file ‘work and non-work’ contains data for investigating the association between sedentary patterns during work and non-work time domain and obesity indicators (N = 671).
(XLSX)

S1 Table Results of single activity* models to estimate the association between sedentary time variables and obesity during whole day (n = 692), work (n = 671) and non-work (n = 671) time domain among blue-collar workers from Danish PHysical ACTivity cohort with Objective measurements (DPhacto). (DOCX)

S2 Table Results of the partition* models to estimate the association between sedentary time variables and obesity during whole day (n = 692), work (n = 671) and non-work (n = 671) time domain among blue-collar workers from Danish PHysical ACTivity cohort with Objective measurements (DPhacto). (DOCX)
