# Supplementary material for: What Is the Effect on Obesity Indicators from Replacing Prolonged Sedentary Time with Brief Sedentary Bouts, Standing and Different Types of Physical Activity during Working Days? A Cross-Sectional Accelerometer-Based Study among Blue-Collar Workers
Source: PLoS One. 2016 May 17;11(5):e0154935. doi: 10.1371/journal.pone.0154935 (PMC4871331; doi:10.1371/journal.pone.0154935)
Supplement: S1 Table — (DOCX) [file pone.0154935.s002.docx]

**S1 Table. Results of single activity* models to estimate the association between sedentary time variables and obesity during whole day (n=692), work (n=671) and non-work (n=671) time domain among blue-collar workers from Danish PHysical ACTivity cohort with Objective measurements (DPhacto).**

| **Variabl**es | **B** | **95%CI** | **P** | **B** | **95%CI** | **P** | **B** | **95%CI** | **P** |
| --- | --- | --- | --- | --- | --- | --- | --- | --- | --- |
|  | Whole day | | | Work | | | Leisure | | |
|  | Waist Circumference | | | | | | | | |
| Total sedentary time | **0.45** | **(0.17 to 0.72)** | **0.00** | 0.06 | (-0.26 to 0.38) | 0.72 | **0.81** | **(0.4 to 1.22)** | **0.00** |
| LB | **0.84** | **(0.52 to 1.16)** | **0.00** | **0.60** | **(0.03 to 1.16)** | **0.04** | **0.91** | **(0.49 to 1.32)** | **0.00** |
| MB | 0.09 | (-0.35 to 0.53) | 0.68 | 0.04 | (-0.46 to 0.53) | 0.88 | 0.26 | (-0.46 to 0.98) | 0.48 |
| SB | **-2.22** | **(-3.14 to -1.29)** | **0.00** | **-1.80** | **(-2.9 to -0.7)** | **0.00** | **-4.98** | **(-7.36 to -2.59)** | **0.00** |
|  | Fat percentage | | | | | | | | |
| Total sedentary time | **0.27** | **(0.12 to 0.41)** | **0.00** | 0.01 | (-0.11 to 0.12) | 0.93 | **0.23** | **(0.08 to 0.37)** | **0.00** |
| LB | **0.46** | **(0.29 to 0.62)** | **0.00** | 0.14 | (-0.06 to 0.35) | 0.18 | **0.30** | **(0.16 to 0.44)** | **0.00** |
| MB | 0.12 | (-0.11 to 0.34) | 0.31 | 0.02 | (-0.16 to 0.19) | 0.85 | -0.05 | (-0.29 to 0.19) | 0.68 |
| SB | **-1.20** | **(-1.67 to -0.72)** | **0.00** | **-0.52** | **(-0.91 to -0.13)** | **0.01** | **-1.84** | **(-2.62 to -1.06)** | **0.00** |
|  | BMI | | | | | | | | |
| Total sedentary time | **0.14** | **(0.04 to 0.24)** | **0.00** | 0.04 | (-0.13 to 0.2) | 0.65 | **0.41** | **(0.2 to 0.62)** | **0.00** |
| LB | **0.30** | **(0.19 to 0.41)** | **0.00** | 0.16 | (-0.14 to 0.47) | 0.29 | **0.54** | **(0.33 to 0.74)** | **0.00** |
| MB | -0.03 | (-0.18 to 0.13) | 0.73 | 0.15 | (-0.1 to 0.41) | 0.24 | -0.06 | (-0.42 to 0.3) | 0.74 |
| SB | **-0.72** | **(-1.04 to -0.39)** | **0.00** | **-0.87** | **(-1.44 to -0.31)** | **0.00** | **-3.36** | **(-4.47 to -2.24)** | **0.00** |

LB= long sedentary bouts (average time/day spent in uninterrupted sedentary bouts >30 min), MB=moderate sedentary bouts (average time/day spent in uninterrupted sedentary bouts >5 and ≤30min), BB= brief sedentary bouts (average time/day spent in uninterrupted sedentary bouts ≤5mins); * adjusted for age, sex, smoking status, alcohol intake, poor dietary patterns and total measured time; estimates in bold are significant at p <0.05.
