# Supplementary material for: What Is the Effect on Obesity Indicators from Replacing Prolonged Sedentary Time with Brief Sedentary Bouts, Standing and Different Types of Physical Activity during Working Days? A Cross-Sectional Accelerometer-Based Study among Blue-Collar Workers
Source: PLoS One. 2016 May 17;11(5):e0154935. doi: 10.1371/journal.pone.0154935 (PMC4871331; doi:10.1371/journal.pone.0154935)
Supplement: S2 Table — (DOCX) [file pone.0154935.s003.docx]

**S2 Table. Results of the partition* models to estimate the association between sedentary time variables and obesity during whole day (n=692), work (n=671) and non-work (n=671) time domain among blue-collar workers from Danish PHysical ACTivity cohort with Objective measurements (DPhacto).**

| Variables | **B(95%CI)** | **P** | B(95%CI) | P | **B(95%CI)** | **P** |
| --- | --- | --- | --- | --- | --- | --- |
|  | Whole day | | Work |  | Non-work | |
|  | Waist Circumference | | | | | |
| Total sedentary time | **0.65(0.24 to 1.07)** | **0.00** | 0.30(-0.24 to 0.83) | 0.27 | **0.87(0.38 to 1.37)** | **0.00** |
| LB | **1.00(0.54 to 1.46)** | **0.00** | 0.63(-0.08 to 1.35) | 0.08 | **0.98(0.47 to 1.48)** | **0.00** |
| MB | **0.64(0.13 to 1.15)** | **0.01** | 0.50(-0.14 to 1.13) | 0.12 | 0.70(-0.06 to 1.46) | 0.07 |
| BB | **-1.60(-2.62 to -0.58)** | **0.00** | **-1.37(-2.56 to -0.18)** | **0.02** | **-2.95(-5.46 to -0.44)** | **0.02** |
|  | Fat percentage | | | | | |
| Total sedentary time | 0.09(-0.11 to 0.29) | 0.36 | -0.08(-0.35 to 0.18) | 0.55 | 0.19(-0.04 to 0.43) | 0.11 |
| LB | **0.24(0.01 to 0.46)** | **0.04** | -0.09(-0.46 to 0.28) | 0.64 | **0.29(0.05 to 0.54)** | **0.02** |
| MB | 0.13(-0.12 to 0.39) | 0.31 | 0.22(-0.11 to 0.55) | 0.19 | -0.03(-0.4 to 0.34) | 0.88 |
| BB | **-1.19(-1.72 to -0.67)** | **0.00** | **-0.82(-1.43 to -0.21)** | **0.01** | **-2.69(-3.87 to -1.5)** | **0.00** |
|  | BMI | | | | | |
| Total sedentary time | **0.17(0.04 to 0.31)** | **0.01** | 0.06(-0.12 to 0.25) | 0.49 | **0.23(0.07 to 0.4)** | **0.01** |
| LB | **0.30(0.18 to 0.45)** | **0.00** | 0.15(-0.1 to 0.41) | 0.24 | **0.30(0.13 to 0.47)** | **0.00** |
| MB | 0.14(-0.04 to 0.32) | 0.13 | 0.17(-0.06 to 0.4) | 0.14 | 0.08(-0.17 to 0.34) | 0.51 |
| BB | **-0.53(-0.89 to -0.16)** | **0.01** | -0.34(-0.77 to 0.08) | 0.11 | **-1.26(-2.1 to -0.43)** | **0.00** |

LB= long sedentary bouts (average time/day spent in uninterrupted sedentary bouts >30 min) , MB=moderate sedentary bouts (average time/day spent in uninterrupted sedentary bouts >5 and ≤30min), BB= brief sedentary bouts (average time/day spent in uninterrupted sedentary bouts ≤5mins); * adjusted for age, sex, smoking status, alcohol intake, poor dietary patterns and all types of physical activity (standing time, walking time, and MVPA); LB, MB, and BB were also mutually adjusted for in the model; estimates in bold are significant at p <0.05. Time spent on each activity except the activity of interest (total sedentary time and LB) is the combined time spent during both domains.
